# Deep learning for electroencephalography emotion recognition

**DOI:** 10.3934/publichealth.2025041

**Published:** 2025-08-06

**Authors:** Hesamoddin Pourrostami, Mohammad M. AlyanNezhadi, Mousa Nazari, Shahab S. Band, Amir Mosavi

**Affiliations:** 1 Department of Computer Science, University of Science and Technology of Mazandaran, Behshahr, Iran; 2 Department of Information Management, International Graduate School of Artificial Intelligence, National Yunlin University of Science and Technology, Douliu, Taiwan; 3 John Von Neumann Faculty of Informatics, Obuda University, Budapest, Hungary; 4 Institute of the Information Society, Ludovika University of Public Service, Budapest, Hungary; 5 Abylkas Saginov Karaganda Technical University, Karaganda, Kazakhstan; 6 Faculty of Economics and Informatics, Univerzita J. Selyeho Komarom, Slovakia

**Keywords:** emotion recognition, electroencephalography, human-computer interaction, machine learning, artificial intelligence, big data, data science, applied AI

## Abstract

This study presents an Electroencephalography (EEG) emotion recognition using a long short-term memory (LSTM)-based method. Our proposed method selects window sizes and overlaps to divide the EEG data into segments, which optimally captures subtle signal changes. A Bidirectional LSTM (BiLSTM) layer is added to standard LSTM layers to better detect forward and backward patterns in the data. By using this dual-layer setup, we aim to improve both the feature extraction and the classification accuracy. The model was tested on the Database for Emotion Analysis using Physiological signals (DEAP) dataset and showed acceptable accuracy across emotional dimensions: arousal (94.0%), liking (98.9%), dominance (95.3%), and valence (99.6%). Our results suggest that the model better supports emotion recognition and has potential for mental health monitoring and adaptive therapy.

## 1. Introduction

**Figure 1.**
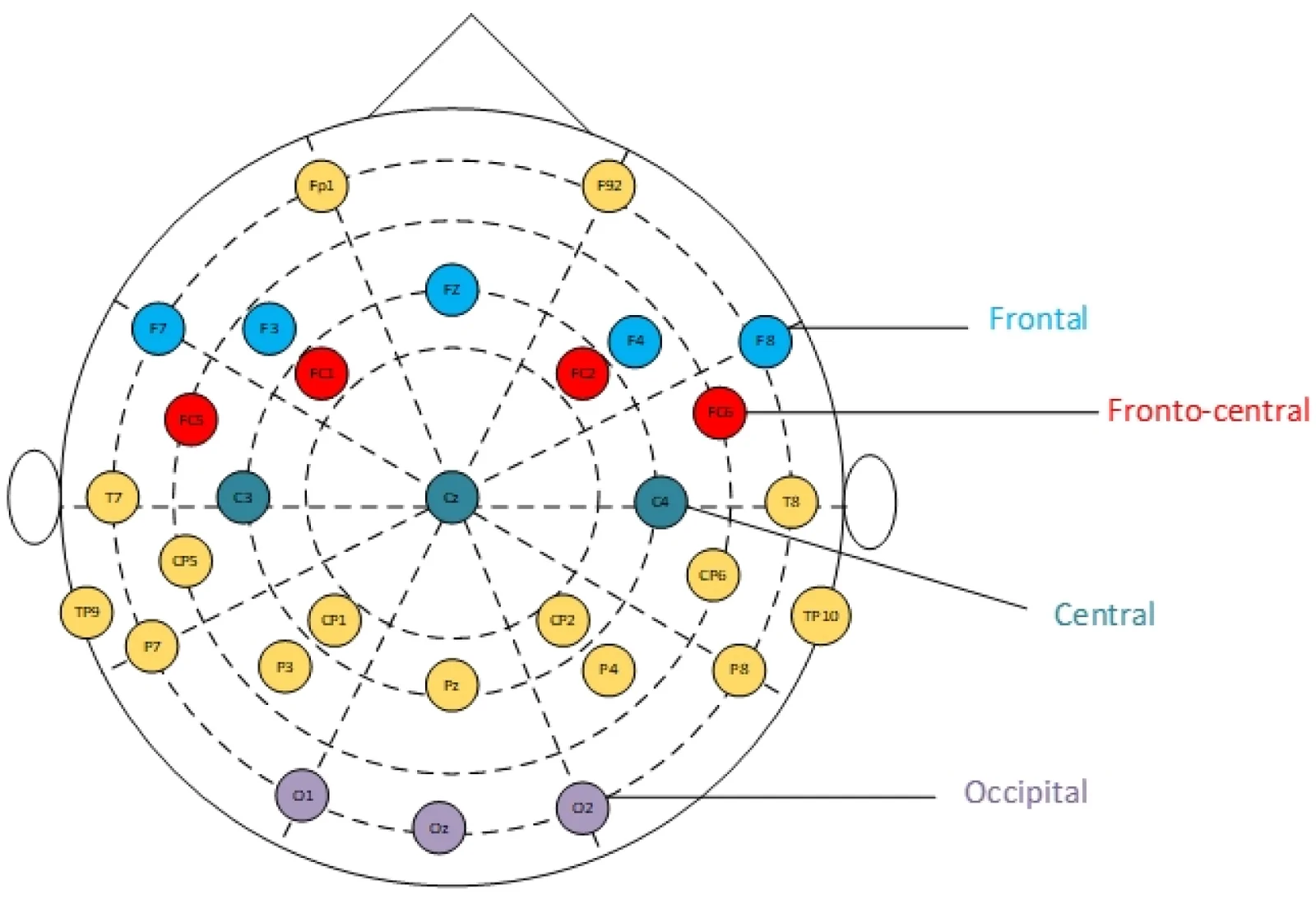
Electroencephalography electrode placement.

Emotion recognition (ER) is the assessment of human emotions and their specification through a range of modalities or channels, which include physiological signals, voice intonations, facial expressions, and brain activity. It is significant in day-to-day activities since it improves an individual's psychological health, which is essential for physical health [1]–[4]. Modern society presents challenges to numerous people regarding their mental health, with the experience of anxiety, stress, and hypertension. The contribution of emotional recognition is helpful to the early identification of these problems and gives enough time to eradicate them ahead of necessary interventions. As a health situation, ER systems can also assist with the identification, diagnosis, and treatment of mental health issues, more precisely, psychosocial interventions, which will reclaim the health situation [5],[6]. The use of ER features can potentially simplify the interactive systems to become simpler regarding Human-Computer Interactions (HCI). Additionally, the systems can become more effective by modifying user interfaces with emotional changes [7],[8]. The ability to recognize the emotions of users demonstrates significant potential for applications in both disciplines and is, therefore, an interesting field of study for scientists and developers alike. However, considering the possibility of faking emotions such as facial expressions and voice intonations, more attention is being given on using emotion recognition by applying several physiological signals such as Galvanic Skin Response, Electromyography, Electrocardiography, Respiration Rate, and Electroencephalogram signals [9]–[11]. Electroencephalography (EEG) is the monitoring of brain activity through the use of electrodes placed on the scalp (see Figure 1).

It emerged as an outstanding technique for ER and the detection of rapid changes in brain activity and dynamics of neurological and emotional processes. The capacity for real-time monitoring has made EEG a powerful method for ER [12]–[15]. EEG signals can be separated into several categories based on the frequency where each one represents a specific activity in the body: delta (0.5–4 Hz), theta (4–8 Hz), alpha (8–13 Hz), beta (13–30 Hz), and gamma (>30 Hz) bands. The Delta band (0.5–4 Hz) is associated with deep, dreamless sleep, anesthesia, and unconscious states caused by low oxygen levels. On the other hand, the Theta band (4–8 Hz) appears during relaxation, positive emotions, and working memory tasks. Alpha waves (8–13 Hz) are linked to resting states and responses to auditory stimuli. The Beta band (13–30 Hz) reflects an active, focused mind, while the Gamma band (above 30 Hz) is related to high-level cognitive processing and the integration of information. Delta waves usually occur with an amplitude of 20–200 µV in the frontal cortex. Theta waves appear and are generally located in the frontal midline with an amplitude of 100–150 µV. Alpha waves mainly occur in the occipital and parietal lobes with an amplitude of 20–100 µV. Beta waves typically appear in the frontal lobe with an amplitude of 5–20 µV. As the last wave of these groups, Gamma waves appear with an amplitude lower than 2 µV and are associated with various sensory and non-sensory cortical networks [16]. Considering the importance of ER nowadays, using EEG signals in this field can enhance the classification of different emotions. However, with all of the advantages of using these signals, some crucial characteristics make this work challenging. EEG signals are typically noisy and are affected by the environment. Additionally, these signals have some artifacts and interferences. This creates a need for accurate preprocessing and analyses. Since EEG contains critical information about the brain's physiological state, splitting this information from noise is sensitive and needs to be carefully cleaned. In this study, to focus more on the novelty of the model architecture, the preprocessed Database for Emotion Analysis using Physiological signals (DEAP) dataset was used. DEAP is a well-known multimodal dataset in ER and is known as a benchmark of physiological analyses. In this dataset, the BioSemi ActiveTwo system recorded 32 channels of EEG data. The EEG signal was provided at a sampling rate of 128 Hz, and the signals were preprocessed to remove noise and artifacts. As the complexity of this field is known, an accurate method needs to be introduced [17],[18]. A novel long short-term memory (LSTM) network architecture was investigated in this research [19]–[21]. This network applies a combination of Bidirectional LSTM (BiLSTM) and LSTM layers to identify and extract complicated patterns from the data. This network also uses a dropout layer to throw away worthless information and help complicated pattern extraction from the data. This novel architecture helps to accurately classify each label and achieve the highest accuracy in comparison with recent works. The rest of the paper is structured as follows: in the second section, the recent investigations are demonstrated; the proposed method workflow is given in Section 3; the dataset and experimental results using the proposed method are emphasized in Section 4; and finally, in Section 5, the study is concluded, and future key points are suggested.

## 2. Related work

In ER, classifying different emotion classes depends on two factors:

- Signal feature extraction; and
- Classifying the extracted features.

Considering the importance of these two factors, the recent research in feature extraction is investigated first. Then, the most applied machine learning methods for ER are introduced.

### 2.1. Feature extraction phase

Trend research on feature extraction from EEG signals can be categorized into three groups: I) time domain, II) frequency domain, and III) time-frequency domain features. Using feature extraction on signal-based datasets is important to convert the raw signals into meaningful information. Sharma et al. [22] proposed the Walsh Hadamard transform, which is a hybrid dual-tree complex wavelet transform used to extract essential time-domain information from biological signals. The developed approach yielded high performance on collected EEG signals by the BCI2000 system with precision, recall, accuracy, specificity, and F1 score of 97.25%, 97.94%, 98.51%, 98.94%, and 97.58%, respectively. Wibawa et al. [23] examined time-domain features, including power spectral density standard, mean, and deviation. Typically, extracting features from signals and selecting channels are based on neuroscientific assumptions. In EEG-based ER, an excessive number of features can reduce the accuracy of classification tasks and increase the computational costs. By feature reduction, the authors improved the accuracy of the ER model. Nath and colleagues [24] aimed to develop an efficient headgear model for on-time emotional state monitoring. Band power, a frequency-domain feature, is an extraction of EEG signals and has been compared across various classifiers for the valence and arousal domains. The proposed approach showed an experimental accuracy of 93.13% on arousal and 94.69% on valence classes. Jiang et al. [25] implemented a privacy-preserving federated learning framework for EEG-based emotion recognition in the context of the Internet of Medical Things (IoMT) and proposed a Gompertz function-based fuzzy rank aggregation method to integrate local client models into a strong global ensemble. Their method achieved a 90.74% overall accuracy on the DEAP and SJTU Emotion EEG Dataset (SEED) benchmarks, while isolated client training suffered a 29.31% accuracy drop, and non-independent and identically distributed (IID) data splits exacerbated the performance by 14.89%, thus illustrating the advantages as well as the difficulties of heterogeneous federated deployment. Saha et al. [26] investigated a new frequency domain common spatial pattern (FCSP) technique to extract features and address limitations, such as being unsuccessful in maintaining separable features between time domain classes, thus leading to erroneous outputs. It involves power spectral density by converting the time-domain EEG signals to identify related event variations in the frequency domain. The classification problem was tested using the proposed approach and demonstrated experiments, and achieved an average accuracy of 91%. Theresia et al. [27] explored the extracted time-frequency domain features from the preprocessed signals of EEG. The method was applied to the DEAP dataset and yielded a recognition rate of 63.75%, thus highlighting the necessity of feature extraction from EEG signals for ER. The efficiencies of Discrete Wavelet Transform (DWT) and Discrete Wavelet Packet Transform (DWPT) were investigated by Ahmad et al. [28]. The study tested different Support Vector Machine (SVM) kernel functions and k-Nearest Neighbors (KNN) distance metrics on subject-dependent and subject-independent approaches. The experiments also utilized the DEAP dataset. The primary difficulty lies in extracting relevant features from EEG signals to achieve an optimal performance. The experimental results indicated the suitability of DWT with the weighted KNN classifier, while DWPT yielded better results with the SVM's linear kernel in accurately classifying EEG signals in a subject-dependent approach. The wavelet-based methods, such as the hybrid Walsh-Hadamard/dual-tree complex wavelet transform by Sharma et al. [22] or the comparative analyses of DWT versus DWPT by Ahmad et al. [28], exhibit the rich capture of temporal dynamics, thereby frequently achieving over a 90% accuracy on BCI2000 and subject-dependent DEAP configurations. Dimensionality and computation efficiency in Wibawa et al.'s [23] work were gained through lean statistical descriptors such as mutual information-based channel selection, though this approach lost precision in capturing frequencies, detail, and overall accuracy. Arousal and valence estimated using simple band power features reached about 93% and 94% accuracy on DEAP, respectively (Nath et al. [24]), though these estimates ignored spatial relationships. Frequency Domain Common Spatial Pattern (FCSP) filtering (Saha et al. [26]) obtained an approximate 91% accuracy, but at an increased computational cost. Standalone spatial-temporal analyses (Theresia et al. [27]) were significantly lower (~63.8%), thus suggesting that without hybrid feature fusion, generalizability across subjects and contexts is limited.

### 2.2. Classification phase

The classification of EEG signals is a significant topic due to this signal's noisy, non-stationary, and high-dimensional nature [29],[30]. A new dynamical graph convolutional neural network (CNN) for the multichannel EEG ER method was proposed by Song et al. [31]. This method models all of the EEG channel features by applying a graph and then classifying the data. In contrast with common graph convolutional neural networks, the proposed approach can strongly find the relationships between EEG channels. Modeling on the SEED dataset showed an average recognition cross-validation accuracy of 90.4% and 79.95% for subject-dependent and subject-independent variables. Moreover, for the DREAMER dataset, average accuracies of 86.23%, 84.54%, and 85.02% were achieved for the valence, arousal, and dominance classes. Feng and colleagues [32] introduced an effective video-level feature organization technique that integrated frequency, spatial domain, and temporal features. Considering the limitation in this field, namely that features from multiple domains are seldom simultaneously analyzed due to the lack of an executable method to organize the features, this investigation demonstrated a deep neural network. It used channel attention convolutional aggregation, which extracts the most relevant emotional state from video-level features. The results showed a well-performed accuracy of 95.80% with a standard deviation of 2.04% on the SEED. This achievement recommends video-level-based features for ER tasks. Algarni et al. [33] proposed an approach based on deep learning techniques for EEG ER signals. It is comprised of several steps, including data selection, feature extraction, feature selection, and classification phases. For the classification stage, a stacked BiLSTM model was employed for human ER. The method demonstrated superior accuracy on the DEAP dataset compared to previous techniques, thereby achieving average accuracies of 99.68% for liking, 99.45% for valence, and 96.87% for arousal. Chakravarthi et al. [34] proposed an automated CNN-LSTM with the ResNet-152 algorithm. This approach aimed to overcome the problems faced by previous researchers. The proposed hybrid deep learning algorithm demonstrated a higher level of accuracy on the SEED-V dataset, thereby achieving 98% compared to existing techniques. In the classification stage, graph convolutional frameworks such as Song et al.'s dynamical graph convolutional network (GCN) [31] capture inter-channel EEG relationships and attain 90.4% (subject dependent) and 79.95% (subject independent) on SEED. However, it may omit finely grained temporal resolution. Attention-based aggregators such as Feng et al.'s channel-attention CNN [32] capture video-level aggregate frequency, spatial, and temporal cues to achieve 95.80% accuracy on SEED, though their batch-level dependence hinders the streaming adaptability. Deep sequential models, particularly the stacked BiLSTM pipeline of Algarni et al. [33], utilize the bidirectional context to surpass 99% accuracy on DEAP's valence and liking, but are overfitting, and cross-dataset validation was not performed. The hybrid architectures of ResNet-LSTM by Chakravarthi et al. [34], which are spatially hierarchical with sequential temporal modeling, struggle with high computations and latency. This produces unbounded latency unfit for real-time workings. In the cross-comparison, the deep recurrent network retains temporal coherence much better than the solo graph and solo CNN methodologies. Even though many contributions have been made to EEG-based emotion recognition, there are still gaps that need to be closed, which motivated the development of this study. The extraction of features from EEG signals is difficult because of their low signal-to-noise ratio, high dimensionality, and tendency to be contaminated. Many machine learning models do not process the complicated temporal dependencies of EEG signals, and this often results in a non-optimal classification accuracy. While recognition performance has certainly been improved by the application of deep learning techniques such as CNNs and standard LSTMs, these deep learning models do not perform well on capturing the multi-scale temporal dependencies of sequential EEG signals. Moreover, some approaches tend to lack segmentation strategies, which can impact the model's ability to extract important information from the data. To fill in these gaps, this study proposes a better segmented hybrid BiLSTM-LSTM network to improve the accuracy and robustness of the model on EEG-based emotion recognition. The main contributions of this study are as follows:

- Development of a novel hybrid BiLSTM-LSTM neural network architecture to capture complex temporal patterns in EEG signals;
- Implementation of a validated approach to determine optimal data segmentation parameters that balance the signal integrity with the computational efficiency while enhancing pattern recognition; and
- Achievement of state-of-the-art performance rates across all four emotion dimensions in the DEAP dataset: 94.0% for arousal, 98.9% for liking, 95.3% for dominance, and 99.6% for valence.

## 3. Proposed method

**Figure 2.**
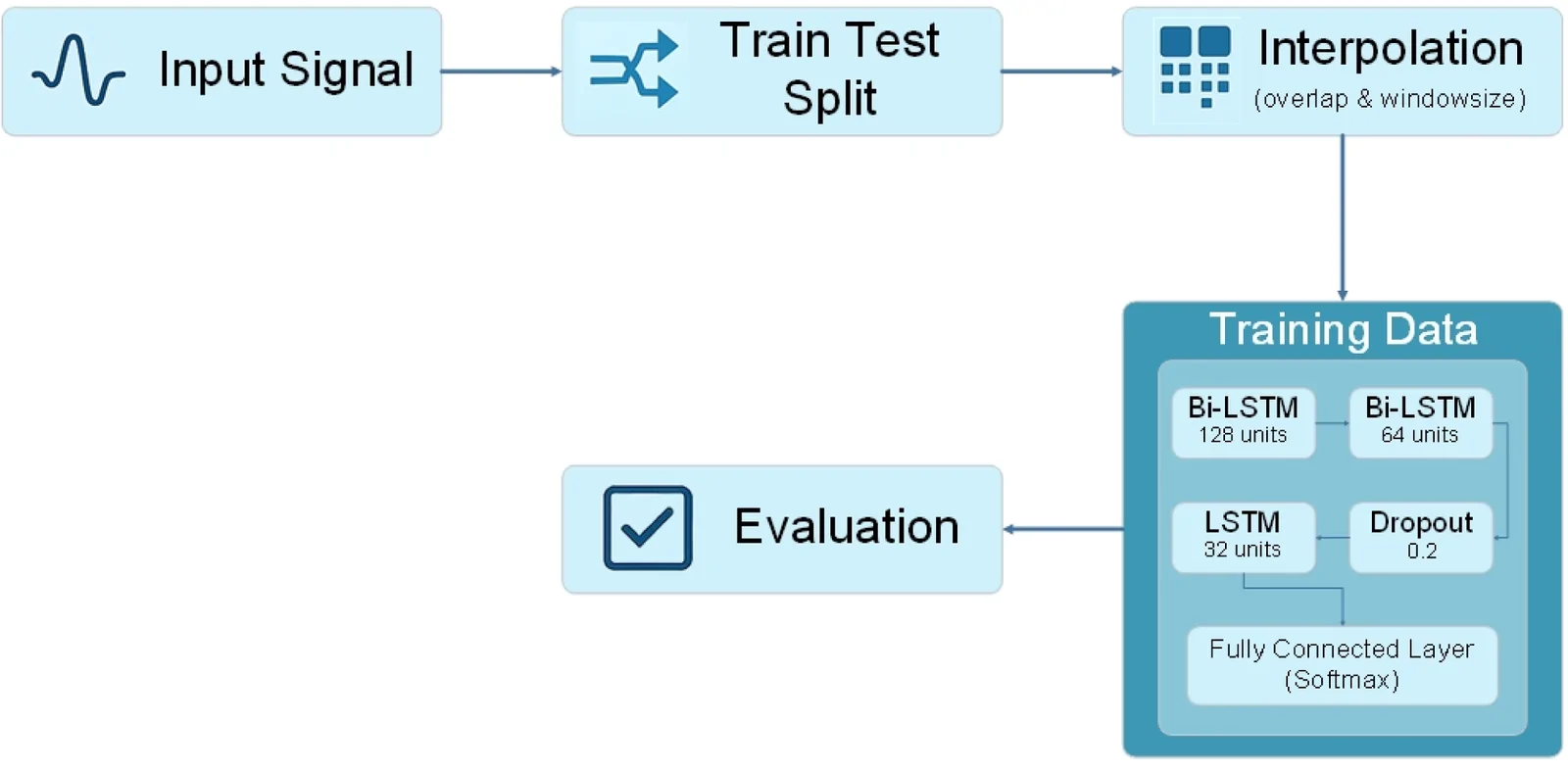
The architecture of the proposed method.

In this section, a new network for human EEG ER signals is proposed. The proposed method is constructed from a novel LSTM architecture on overlapping windowed signals by considering three main factors: I) finding the optimal window, II) finding the optimal overlap, and III) designing the construction of the LSTM architecture (see Figure 2). Thus, the proposed model can classify different human emotions.

### 3.1. Finding the optimal window

Considering that existing complex and different patterns in EEG signals make the work more sophisticated, the data needs to be more scalable and simpler. This process helps the model find the data patterns faster and more accurately, and allows the model to learn features from its temporal evolution [35]–[37]. In this research, each row of the signal is constructed from 8064 data points which correspond to the EEG signal sampled at 128 Hz over 63 seconds. Since choosing the window size (N) is a crucial parameter that balances the trade-off between the signal's length and computational cost, the number of splits was accurately chosen. In each iteration, this window size was tested on the data using the network architecture, and the achieved result was saved to compare with the next result and set as the optimal parameter if it had the highest result in all iterations.

### 3.2. Finding the optimal overlap

Each EEG signal contains important features of brain activity. Additionally, the EEG signals may contain common pieces of information. Recognizing these pieces of information can influence the model's efficiency and eventually simplify the data to find patterns and detect the class of the data. Besides using segmentation on each row and finding an optimal window size, making overlaps between these processed windows is crucial and helps to identify common information and patterns more easily. Similar to Section 3.1, this process starts with initializing an overlap. Opposite to the size of the optimal window, the achieved number is the number of samples that don't overlap with each other, that is, if the number of windows is N and O is the representative of overlap, then the number of samples that overlap with each other is N-O. This process makes the data simpler and more appropriate for the network and eventually enhances the achieved results before using the combination of these two techniques.

### 3.3. Classification using a novel LSTM architecture

Despite the combination of the optimal window size and overlap, it is very important to use a reliable network to realize suitable data. An appropriate network can process the data very well and use it for remarkable data classification conclusions. Because the EEG data is complex and sophisticated enough, using every simple and trend method cannot produce good achievement, and it has pushed scientists to use deep learning methods [30],[38]. This study applies a recurrent neural network (RNN) to categorize different emotion classes. The network receives preprocessed EEG data as the input, which has been segmented into overlapping windows. These windows are provided as sequential inputs to the network, making it more appropriate for the model to learn the temporal structure of the EEG signals. The proposed network architecture is constructed from several layers: BiLSTM layers, a dropout layer, an LSTM layer, and a fully connected layer. This architecture uses the strengths of BiLSTM and LSTM layers to capture both bidirectional and unidirectional temporal dependencies in EEG signals, alongside a dropout layer to enhance the generalization. LSTM is one of the powerful recurrent methods that is used in the ER field and EEG signal classification to solve difficult pattern problems. It is capable of learning long-term dependencies, which is important for tasks when the current prediction is conditional on previous inputs. LSTM puts together a set of gates—the input gate, forget gate, and output gate—to overcome gradient problems, which are traditional problems of RNNs. These gates assist in modulating the process of information in the network and allow it to either retain or forget information. BiLSTM, an advanced type of LSTM network, was designed to capture complex temporal dependencies in sequential data. This network uses dual processing (forward and backward direction) to rely on the entire sequence when predicting instead of considering only the past context. In the forward phase, the sequence input data is processed from the beginning to the end to extract past information. Simultaneously, the backward phase processes information in reverse from the end to the beginning to capture future context. The first layer of the proposed network contains a BiLSTM layer with 128 units, which are responsible for capturing the initial temporal features. To refine the achieved information from the previous stage, another BiLSTM layer with 64 units is used to extend the first layer. This process helps to understand the sequence more comprehensively and utilize the data more easily. To avoid overfitting, a regularization technique named the dropout layer is used after two sequence BiLSTM layers. A fraction of the input units is set to zero by this layer during the training to ensure that the network does not become overly reliant on any particular neurons. After the dropout layer, a single-directional LSTM layer with 32 units is applied. This layer is used to focus on the main temporal features from the previous layers. By reducing the number of units in this layer, we aimed to fix the features and prepare them for the final classification stage. The final layer in the architecture is a fully connected layer, which is employed as a dense classifier. This layer considers the features extracted by the LSTM layers and outputs a probability distribution over the possible emotional classes. The SoftMax activation function is used in this layer to ensure that the output is interpretable as the probability of each class.

## 4. Experiments and results

In this section, the experimental results are given. First, the used dataset is introduced. Then, the metrics for measuring the model's performance are discussed. Finally, the achieved result on the introduced dataset using the proposed method is investigated, and a comprehensive comparison between our proposed method and the trend high-performance approaches is reported.

### 4.1. Dataset

In this study, the DEAP dataset [39] is used to emphasize the remarkability of the proposed method. DEAP is a dataset for multi-modal emotion analyses using EEG signals. It is used in ER and is famous as a benchmark in HCI. This dataset was collected from the EEG and peripheral physiological signals of 32 participants to analyze human affective states. To use the data, 40 one-minute videos were shown to participants. After watching the videos, the participants rated each video separately in the scope of the levels of arousal, valence, liking, and dominance, with an integer score between 1 and 9. In this process, 32 electrodes were placed on the international system, and 8 other peripheral physiological signals were recorded. Each trial's data consists of 8064 data points per channel, and covers 63 seconds of recording (including a 3-second pre-trial baseline). In this paper, we divided the dataset into 80% to train the network and 20% to evaluate the model. In addition, the threshold of five was determined for each label to convert them into binary classes (ratings lower than five are assigned to negative and otherwise assigned to positive). Additionally, the ratio of samples is 40%–60%, 47%–53%, 35%–65%, and 7%–93% for the arousal, valence, dominance, and liking classes, respectively.

To further assess the reliability of the proposed method and investigate various issues in the ER field, the SEED [40],[41] is utilized. The SEED is a benchmark dataset for emotion recognition and is constructed from EEG signals recorded from 15 participants while watching 15 Chinese clips (~4 minutes each). The recordings were collected on three different days for each participant and categorized as either positive (1), neutral (0), or negative (−1).

### 4.2. Evaluation metrics

To monitor the proposed method's performance, it is crucial to use appropriate metrics. In this investigation, considering to complexity of the problem, accuracy, precision, recall, and f1-score are applied to comprehensively review the model's efficiency. The essential variables of the introduced metrics must be calculated before, are demonstrated in Table 1.

**Table 1.** Essential variables of evaluation metrics.

Variable	Name	Description
TP	True Positive	The number of correct identifications of positive instances as positive
FP	False Positive	The number of incorrect identifications of negative instances as positive
TN	True Negative	The number of correct identifications of negative instances as negative
FN	False Negative	The number of incorrect identifications of positive instances as negative

As the first and most used metric in recent research, accuracy plays a main role in measuring the ability of the model in classification tasks. It counts the number of correct predictions as true positives and true negatives out of all of the instances in both classes of the data. In a binary-class classification, this metric is calculated by the following formula:

$$Accuracy=\frac{TP+TN}{TP+TN+FP+FN}$$ (1)

Precision is frequently used metric, where the number of correct and positive predictions is important and influences the reliability of the approach. It calculates the ratio of true positives out of all of the instances that are predicted positive by the following formula:

$$Precision=\frac{TP}{TP+FP}$$ (2)

Recall is used to measure the model's performance in correctly predicting positive instances out of all of the positive cases in the data. Recall is calculated using the following formula:

$$Recall=\frac{TP}{TP+FN}$$ (3)

Despite using precision and recall, it is also essential to use a metric that balances these two metrics. The F1-score combines precision and recall together to measure the trade-off between these metrics. To calculate the F1-score, the following formula is used:

$$F1-score=2\times \frac{precision\;\times recall}{precision+recall}$$ (4)

A Receiver Operating Characteristic (ROC) curve is a graphical tool for plotting the sensitivity rate against the false positive rate. This metric explains how well the model separates positive and negative classes without committing to any single threshold. This tool is constructed from the X-axis, which shows the false positive rate, and the Y-axis, which shows the true positive rate, and ranges between 0 and 1. These axes can be calculated using the following formulas:

$$FPR=\frac{false\;positives}{false\;positives+true\;negatives}$$ (5)

$$TPR=\frac{true\;positives}{true\;positives+false\;negatives}$$ (6)

A confusion matrix is a 2 × 2 table that shows the number of correct and incorrect predictions of each class. It demonstrates how well the model's predictions match with actual values. This tool not only measures the overall accuracy but also the type of errors the model made.

### 4.3. Results

In this section, the experimental results of the dataset using the proposed method are shown in three subsections. In subsection 1, the different window sizes are demonstrated, and the optimal window size is chosen. A comparison between diverse overlap sizes is given in subsection 2. Finally, in subsection 3, the achieved results on all four labels using the optimal window and overlap size are shown. Additionally, in this section, a comparison between the recent high-performance approaches and our method is discussed, and for further assessments, the proposed approach is applied to the SEED. In this investigation, we determined the Adam optimizer with a *learning rate* = 0.001 for all of the phases. In all of the experiments, a *batch size* = 32 is applied to the dataset, and the training process is run over five epochs using an Intel E5-2690 CPU, 40 GB DDR4 RAM, with MATLAB R2022a on a Windows 10 Enterprise server. In phase one, windows with different sizes are experimented on all labels (Table 2).

**Table 2.** The efficiency of different window sizes using the proposed method (overlap size = 6 samples).

Emotion	Window size/metric	4000 samples	3500 samples	3000 samples	2500 samples	2000 samples	1500 samples	1000 samples
Valence	Acc	0.9962	0.9057	0.8366	0.8235	0.8210	0.8180	0.8110
	Pre	0.9970	0.8555	0.9051	0.8500	0.8365	0.7978	0.7839
	Recall	0.9944	0.9407	0.7862	0.7954	0.8039	0.8199	0.8108
	F1	0.9957	0.8961	0.8414	0.8312	0.8579	0.7786	0.7981
Arousal	Acc	0.9398	0.8682	0.8497	0.8394	0.8132	0.7726	0.7699
	Pre	0.9519	0.8538	0.8982	0.8465	0.8771	0.8553	0.8649
	Recall	0.9474	0.9188	0.8575	0.8532	0.8001	0.7996	0.7764
	F1	0.9496	0.8851	0.8774	0.8694	0.8617	0.8313	0.8183
Dominance	Acc	0.9528	0.9266	0.8609	0.8590	0.8414	0.8371	0.8322
	Pre	0.9492	0.9337	0.9186	0.9018	0.8837	0.8573	0.8509
	Recall	0.9767	0.9498	0.8725	0.8816	0.8763	0.8849	0.8839
	F1	0.9628	0.9417	0.8949	0.8796	0.8711	0.8664	0.8670
Liking	Acc	0.9892	0.9817	0.9773	0.9717	0.9665	0.9588	0.9557
	Pre	0.9891	0.9953	0.9970	0.9937	0.9976	0.9953	0.9988
	Recall	0.9996	0.9856	0.9795	0.9737	0.9669	0.9598	0.9565
	F1	0.9943	0.9904	0.9882	0.9813	0.9742	0.9829	0.9772

Considering the necessity of making overlaps between the windows (the overlap size is fixed in this phase), which makes more similar instances in the data, it is demonstrable that increasing the number of segments has a reverse influence. With the increase in the number of windows and the existing overlap between two continuous windows, the model can be interrupted from the main features of the data and pay attention to just the similar secondary features created by the overlap. Thus, increasing the number of segments increases the overlaps between windows and eventually makes recognizing these windows more difficult. To avoid these problems by perfectly separating the main features of each row and avoiding biased decisions, the number of segments is chosen experimentally to be two in this phase, which is neither larger nor smaller.

**Figure 3.**
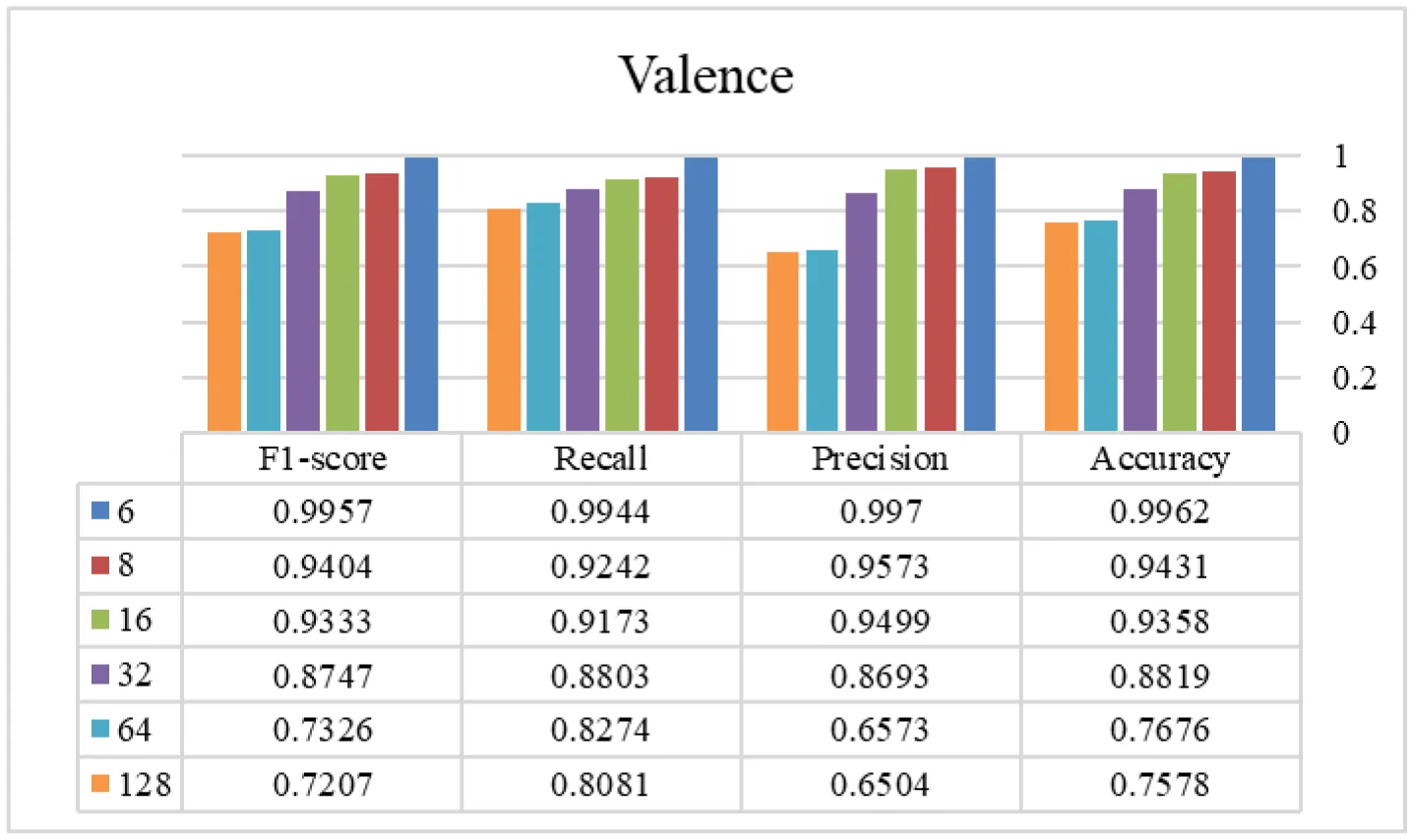
The efficiency of different overlap sizes using the proposed method on the valence class (window size = 4032 samples).

**Figure 4.**
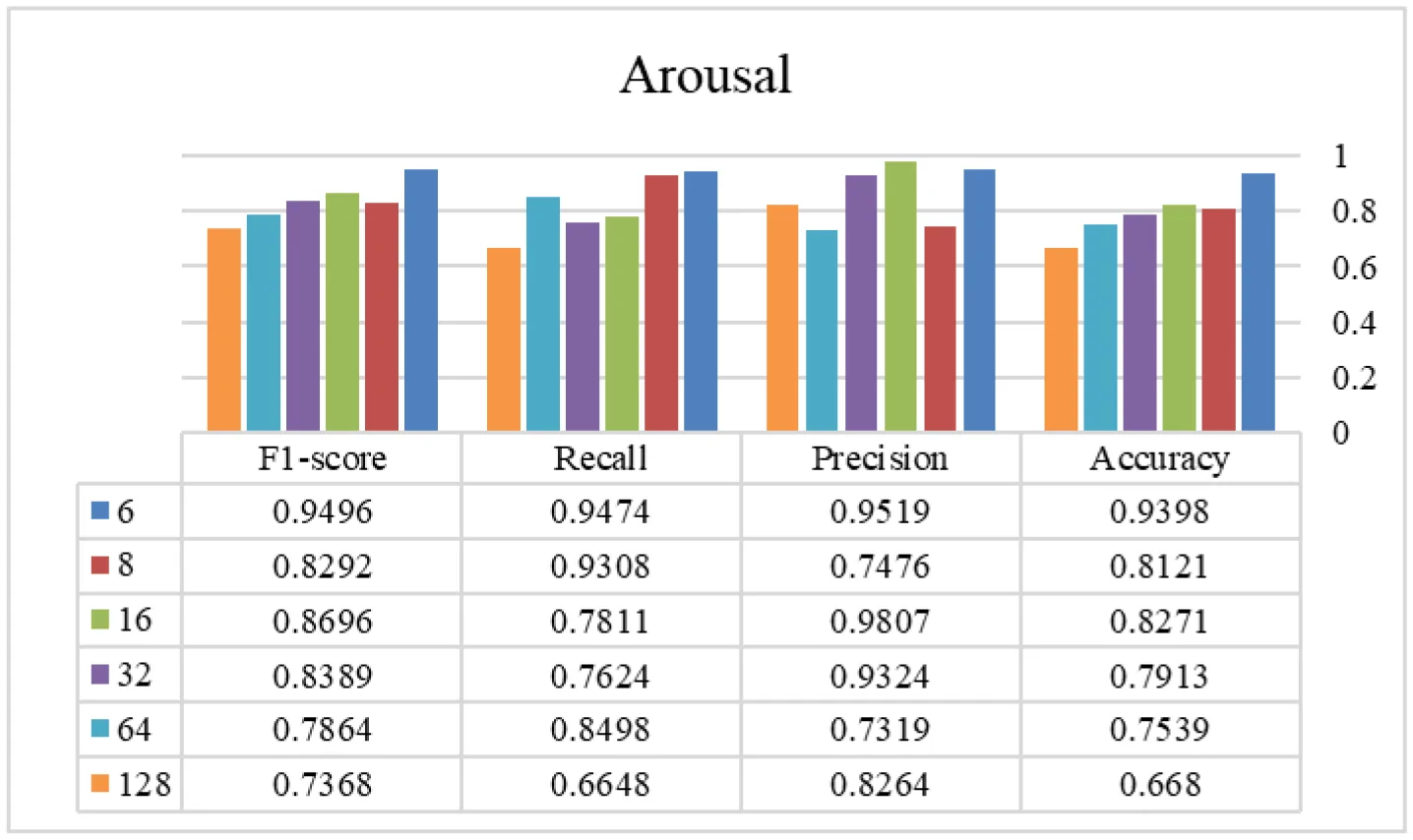
The efficiency of different overlap sizes using the proposed method on the arousal class (window size = 4032 samples).

**Figure 5.**
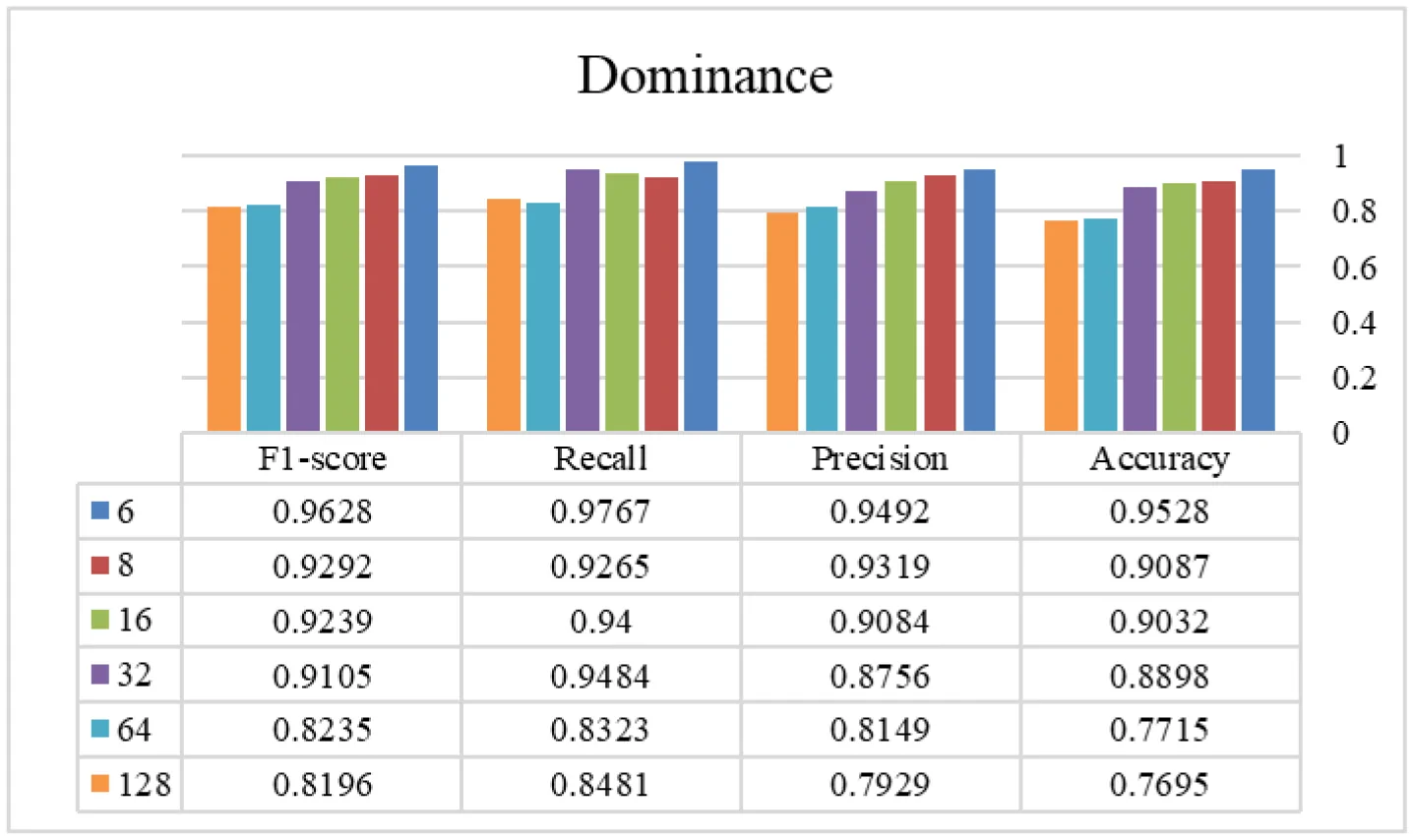
The efficiency of different overlap sizes using the proposed method on the dominance class (window size = 4032 samples).

**Figure 6.**
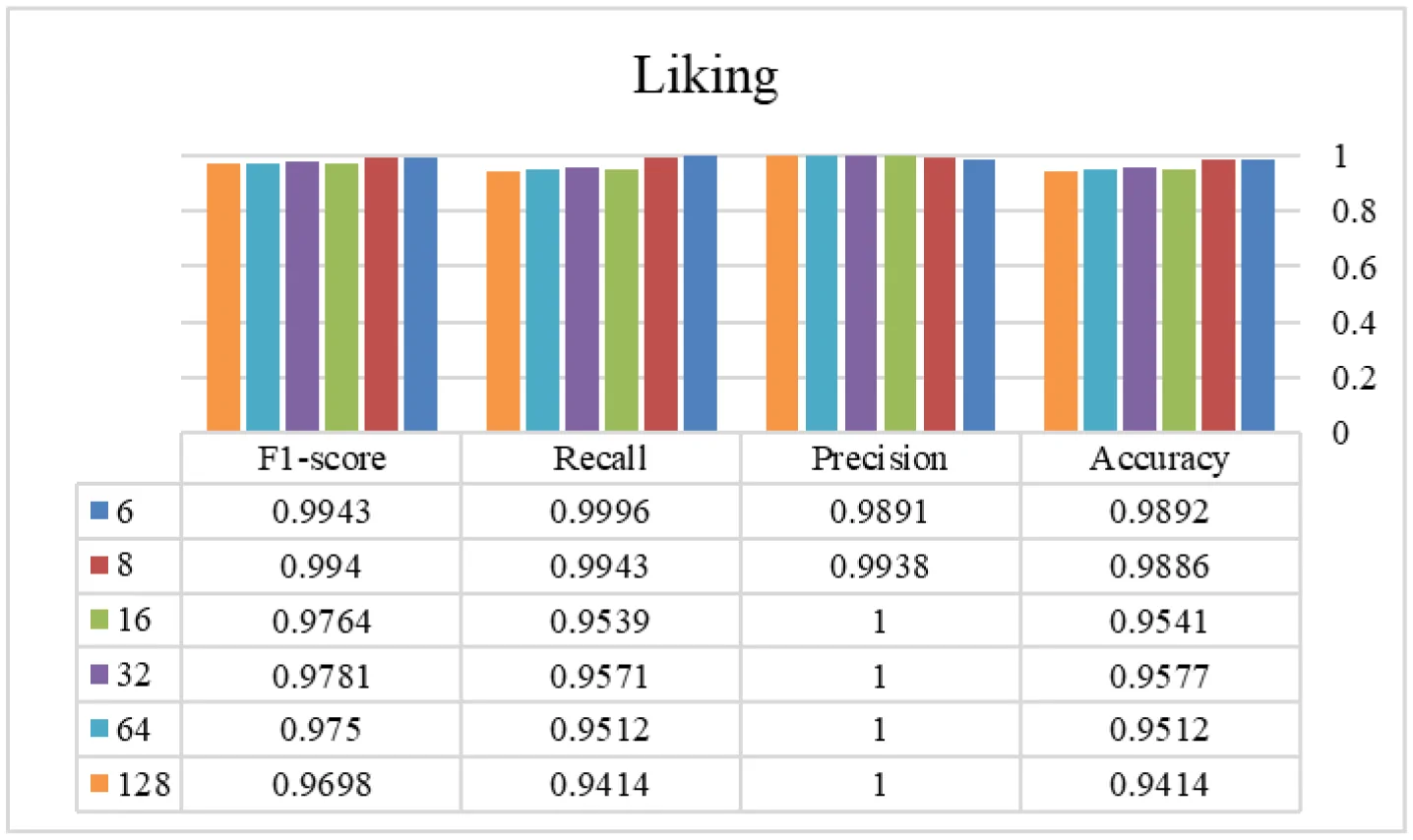
The efficiency of different overlap sizes using the proposed method on the liking class (window size = 4032 samples).

In phase two, the optimal window size from the previous stage is selected and used to find the appropriate overlap size. Figures 3–6 show the tryout of the proposed method using the optimal segment number and different overlap sizes on the introduced dataset. Since the overlap number in this study is the number of instances that are not the same in two continuous windows, it can be demonstrated that increasing this number influences biased decisions and increments it. It should be mentioned that as much as we increase this number and make non-equal instances between windows, the performance of the model will decrease and cause the model's weakness. As the window sizes are chosen as two in the previous stage, it is better to keep these two windows more similar to each other and select the overlap number as small as possible. Experimentally, by choosing 6 as an overlap size, we can achieve the highest results of the proposed approach in all of the evaluation metrics. Overall, using the proposed architecture, the best-achieved performance between the experimented window and overlap sizes is a window size of 4032 and an overlap size of 6, which is shown in Table 3. Additionally, we investigated different combinations of Bi-LSTM and LSTM layers to demonstrate the influence of each layer in this table. As shown, changing/removing each one of the layers can cause a decrease in the reliability of the framework and eventually a lower performance. By choosing appropriate layers for the network and integrating with the overlap and window parameters, the model's ability to recognize different classes is enhanced and suggests a reliable approach in tasks with this level of complexity. According to the sensitivity of the problem and its crucial development in real-world applications, we assessed our proposed method using a 5-fold cross-validation, as shown in Table 4. This table shows that our proposed method has a standard deviation between 0.004 and 0.022, which proves its stability in all of the criteria. Finally, we provided a comprehensive review of our method against recent high-performance studies presented in Table 5.

**Table 3.** The performance of the proposed method.

Approach	Metrics	Arousal	Valence	Dominance	Liking
Proposed method	Accuracy	0.940	0.996	0.953	0.989
	Precision	0.952	0.997	0.949	0.989
	Recall	0.947	0.994	0.977	1.000
	F1-score	0.950	0.995	0.963	0.994
Bi-LSTM (128) + dropout (0.5) + LSTM (64)	Accuracy	0.902	0.944	0.931	0.978
	Precision	0.878	0.973	0.913	0.952
	Recall	0.902	0.914	0.931	1.0
	F1-score	0.889	0.943	0.922	0.975
Bi-LSTM (128) + dropout (0.5) + Bi-LSTM (64)	Accuracy	0.917	0.945	0.928	0.968
	Precision	0.895	0.895	0.904	0.932
	Recall	0.917	0.989	0.933	0.999
	F1-score	0.906	0.940	0.918	0.964
LSTM (128) + dropout (0.2)	Accuracy	0.763	0.811	0.872	0.898
	Precision	0.714	0.680	0.840	0.875
	Recall	0.762	0.898	0.872	0.895
	F1-score	0.738	0.774	0.856	0.884

**Table 4.** The assessment of the proposed method using 5-fold cross-validation.

Metrics	Arousal	Valence	Dominance	Liking
Accuracy	0.943 ± 0.005	0.979 ± 0.014	0.949 ± 0.005	0.978 ± 0.004
Precision	0.934 ± 0.012	0.985 ± 0.008	0.936 ± 0.008	0.983 ± 0.005
Recall	0.945 ± 0.005	0.970 ± 0.022	0.953 ± 0.013	0.989 ± 0.007
F1-score	0.939 ± 0.008	0.977 ± 0.014	0.944 ± 0.008	0.986 ± 0.006

**Table 5.** Comparison of the proposed method and existing high-performance approaches on the DEAP dataset (accuracy).

**Ref.**	**Year**	**Method**	**Arousal**	**Valence**	**Dominance**	**Liking**
[42]	2016	SVM + RVM	0.68	0.65	0.63	0.67
[29]	2017	LSTM	0.856	0.854	-	0.880
[11]	2018	ANN	0.821	0.820	-	-
[38]	2018	KNN	0.929	0.928	-	-
[30]	2019	DNN	0.613	0.625	-	-
[43]	2019	Merged LSTM	0.838	0.849	0.844	0.807
[36]	2020	LSTM	0.852	0.842	-	-
[37]	2020	RACNN	0.971	0.966	-	-
[44]	2021	CNN + SVM	0.777	0.766	-	-
[45]	2021	BiDCNN	0.974	0.944	-	-
[21]	2022	LSTM	0.908	0.909	-	-
[46]	2022	DNN	0.612	0.625	-	-
[18]	2023	ACRNN	0.933	0.937	-	-
[47]	2023	SparseDGCNN	0.666	0.646	-	-
[48]	2023	MTLFuseNet	0.833	0.804	-	-
[49]	2024	SVM	0.708	0.760	-	-
[50]	2024	EPNNE	0.760	0.753	-	-
[51]	2024	MI-EEG	0.565	0.577	-	-
[52]	2024	DG-JCA	0.685	0.697	-	-
[53]	2024	DA-CapsNet	0.752	0.736	-	-
[54]	2025	SIMA-CNNBLS	0.844	0.836	-	-
[54]	2025	GIMA-CNNBLS	0.846	0.837	-	-
**Proposed Method**	**-**	**BiLSTM-LSTM**	**0.940**	**0.996**	**0.953**	**0.989**

**Figure 7.**
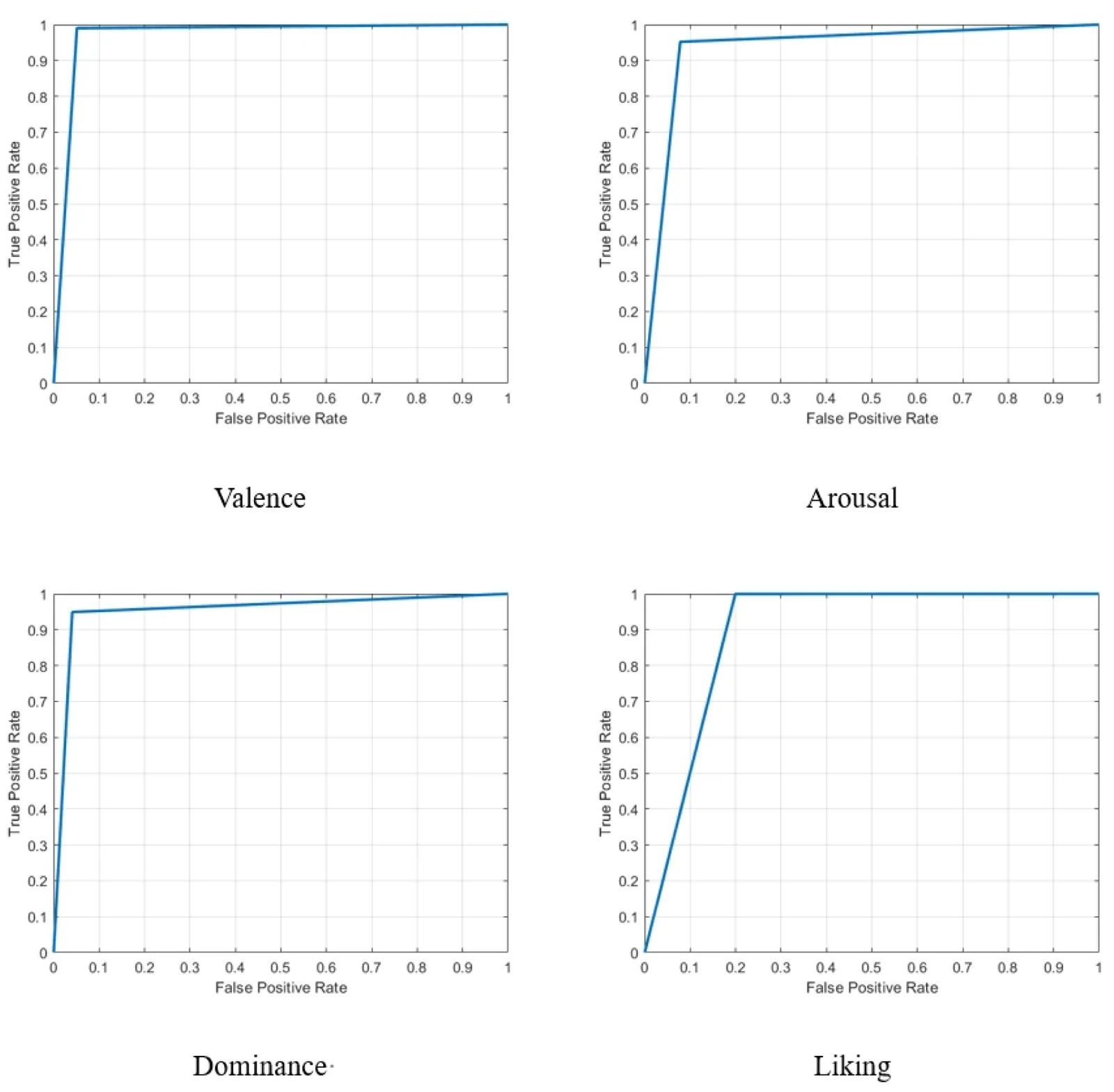
The ROC curve of predicted emotion labels.

**Figure 8.**
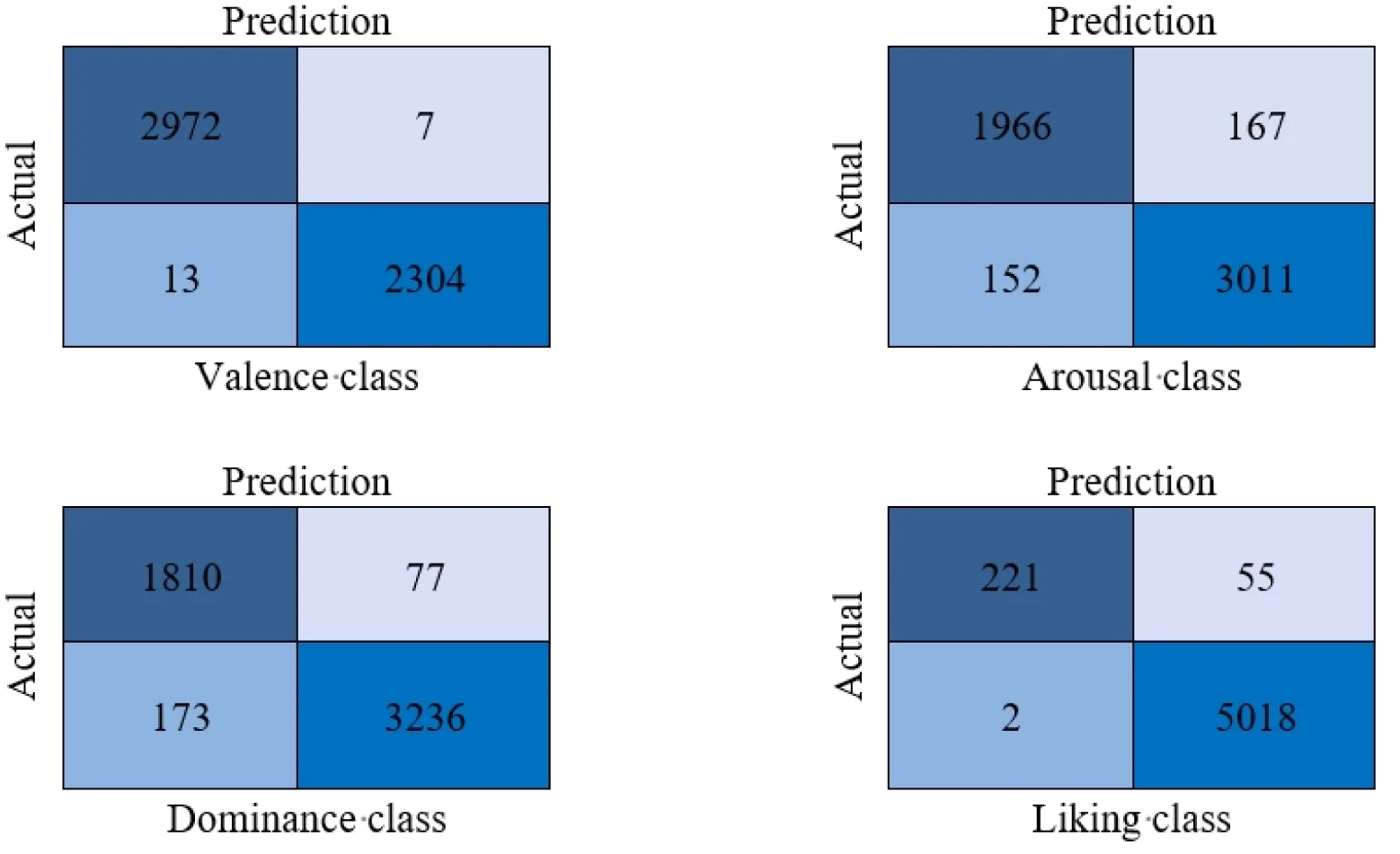
The confusion matrices of emotion classes for the DEAP dataset.

The recent approaches are separated into machine learning (ML) and deep learning (DL) methods. As expected, almost all of the ML methods had a poor performance in recognizing the classes of the data, which clearly shows the hardness of the problem. Contrary to the ML methods, most DL methods have successfully detected almost 80% of instances of a class and show the ability of neural networks in complicated problems. Additionally, with deeper insight, it can be seen that networks with the base of LSTM and Bidirectional networks, such as [37],[45], achieved a high performance in recognizing the classes of the data. Thus, these types of networks are recommended for tasks like this. As mentioned in Section 3, our approach was constructed from integrative BiLSTM and LSTM methods, which are some of the most reliable methods in recent research. By putting appropriate different layers together, our approach gained the highest performance in recent investigations in all four labels, thereby considering all of the evaluation metrics, which shows the ability of our method in all sides of this problem. Figure 7 demonstrates the ROC curves for the valence, arousal, dominance, and liking classes. As shown, the proposed method was completely successful in separating high versus low states in all of the classes, while the framework had the minimum misclassification. Additionally, to demonstrate the ability of the proposed method, the confusion matrix of each class is provided in Figure 8. The confusion matrix shows the number of correct predictions and compares it with the number of misclassifications. As shown, the method has a high ratio of correct predictions in both the positive and negative samples, even in classes such as dominance and liking that suffer from the imbalance problem, which proves it as a reliable framework for the future. The proposed approach achieved an accuracy of 0.94% in the arousal class and an accuracy of 0.99% in the valence class, which are the hardest labels of this data; this shows the ability of the proposed approach to recognize different classes very well and suggests using networks like this in this type of investigation. Moreover, while our framework captures both past and future temporal dependencies, other methods such as CNN-based schemes, the Residual Attention Convolutional Neural Network (RACNN) [37], the Bi-Hemisphere Discrepancy Convolutional Neural Network (BIDCNN) [45], and ResNet-152 of Chakravarthi et al. [34] failed to classify some emotion labels. Additionally, by converting the data sequences to overlapping segments, our method overtook the mentioned approaches, thus leading to a faster convergence and eventually a faster training time. Although our proposed method offers significant advantages, lightweight architectures such as CNNs or hybrid CNN-LSTMs require fewer computational and memory resources, which helps in real-time or resource-constrained developments.

To demonstrate the reliability of the proposed framework, we applied it to the SEED. Table 6 shows the performance of the proposed method on this dataset and compares the method with recent high-performance research. As shown, our framework was successful in separating the classes into positive, neutral, and negative categories on the SEED, thus achieving a higher performance and a less complex approach compared to recent investigations.

**Table 6.** The comparison of the performance of the proposed method on the SEED dataset (accuracy).

**Ref.**	**Year**	**Method**	**Accuracy**
[55]	2018	SVM	0.833
[31]	2018	DGCNN	0.797
[56]	2018	RF	0.928
[57]	2019	SVM	0.890
[58]	2019	GELM	0.880
[59]	2020	SRU	0.831
[60]	2020	Windowing CNN	0.865
[61]	2020	LSTM	0.909
[62]	2020	STFT CNN	0.905
[44]	2021	CNN	0.884
[63]	2022	RGNN	0.853
[46]	2022	Ensemble ML	0.813
[64]	2024	DAEST	0.881
[65]	2024	DGCNN	0.899
**Proposed method**	**-**	**BiLSTM-LSTM**	**0.957**

## 5. Conclusions

An architecture for EEG-based emotion recognition using an enhanced LSTM was presented. The model combines optimized data segmentation with a tailored neural network structure. We showed that the model achieved acceptable performances across all four emotion categories in the DEAP dataset. The use of Bi-LSTM and LSTM layers proved effective in processing physiological EEG signals. A window size of 4032 samples and an overlap of 6 samples significantly improved the classification accuracy. Our model demonstrated accuracy in a challenging task, which achieved 94.0% for arousal and 99.6% for valence. A comparative analysis with recent high-performance models confirmed its robustness and reliability. Future research may apply this approach to other EEG datasets and explore its use in real-time systems, e.g., [66]. Additionally, enhancing the model's interpretability could reveal valuable insights into the neural mechanisms of emotion. Overall, this work advances EEG-based emotion recognition and offers a promising tool for affective computing and mental health applications.

## Use of AI tools declaration

After preparation of the first draft, large language models had been used to improve the readability and language of the manuscript and to remove the potential typos. We carefully reviewed and edited the content as needed and take full responsibility for the content.
